# The association between patients’ expectations and experiences of task‐, affect‐ and therapy‐oriented communication and their anxiety in medically unexplained symptoms consultations

**DOI:** 10.1111/hex.12854

**Published:** 2018-12-30

**Authors:** Juul Houwen, Bas J. E. Moorthaemer, Peter L. B. J. Lucassen, Reinier P. Akkermans, Willem J. J. Assendelft, Tim C. olde Hartman, Sandra van Dulmen

**Affiliations:** ^1^ Department of Primary and Community Care Radboud University Medical Center Radboud Institute for Health Sciences Nijmegen The Netherlands; ^2^ Radboud Institute for Health Sciences IQ Healthcare Radboud University Medical Center Nijmegen The Netherlands; ^3^ NIVEL (Netherlands Institute for Health Services Research) Utrecht The Netherlands; ^4^ Faculty of Health and Social Sciences University of South‐Eastern Norway Drammen Norway

**Keywords:** anxiety, consultation, doctor‐patient communication, expectations, general practice, medically unexplained symptoms

## Abstract

**Background:**

It is unknown whether patients with medically unexplained symptoms (MUS) differ from patients with medically explained symptoms (MES) regarding their expectations and experiences on task‐oriented communication (ie, communication in which the primary focus is on exchanging medical content), affect‐oriented communication (ie, communication in which the primary focus is on the emotional aspects of the interaction) and therapy‐oriented communication (ie, communication in which the primary focus is on therapeutic aspects) of the consultation and the extent to which GPs meet their expectations.

**Objective:**

This study aims to explore (a) differences in patients’ expectations and experiences in consultations with MUS patients and patients with MES and (b) the influence of patients’ experiences in these consultations on their post‐visit anxiety level.

**Study design:**

Prospective cohort.

**Setting:**

Eleven Dutch general practices.

**Measurements:**

Patients completed the QUOTE‐COMM (Quality Of communication Through the patients’ Eyes) questionnaire before and after the consultation to assess their expectations and experiences and these were related to changes in patients’ state anxiety (abbreviated State‐Trait Anxiety Inventory; STAI).

**Results:**

Expectations did not differ between patients with MUS and MES. Patients presenting with either MUS or MES rated their experiences for task‐related and affect‐oriented communication of their GP higher than their expectations. GPs met patients’ expectations less often on task‐oriented communication in MUS patients compared to MES patients (70.2% vs 80.9%; *P* = ˂0.001). Affect‐oriented communication seems to be most important in reducing the anxiety level of MUS patients (β −0.63, 95% Cl = −1.07 to −0.19).

**Discussion:**

Although the expectations of MUS patients are less often met compared to those of MES patients, GPs often communicate according to patients’ expectations. Experiencing affect‐oriented communication is associated with a stronger reduction in anxiety in patients with MUS than in those with MES.

**Conclusion:**

GPs communicate according to patients’ expectations. However, GPs met patients’ expectations on task‐oriented communication less often in patients with MUS compared to patients with MES. Experiencing affect‐oriented communication had a stronger association with the post‐consultation anxiety for patients with MUS than MES.

## 1 INTRODUCTION

Medically unexplained symptoms (MUS) are symptoms for which, after a thorough history taking, physical and additional investigations, no pathological cause can be found.^1^ As 3%‐11% of consultations in primary care concern MUS, GPs often face patients with MUS.^2^, ^3^, ^4^ MUS represent a variety of symptoms like headache, dizziness, fatigue and abdominal discomfort. Patients with MUS often ask for extra time, emotional support and empathy when they consult a GP.^5^ Furthermore, they expect to receive an explanation and a diagnosis.^6^, ^7^ Salmon et al^5^ showed that expectations of patients with MUS differ from expectations of patients with medically explained symptoms (MES). For example, patients with MUS seek significantly more emotional and moral support. However, they do not seek more often for explanation, reassurance or somatic intervention than patients with MES.^5^

Patients with severe MUS are often dissatisfied with the care they receive.^8^, ^9^, ^10^ Salmon et al^11^ showed that MUS patients experienced the explanations of most GPs as being at odds with their own thinking. This is line with the results of another study in which patients with chronic fatigue syndrome were dissatisfied with the quality of medical care received during their illness as they received an unacceptable psychiatric diagnosis for their symptoms.^6^ Johansson et al^12^ described that women with musculoskeletal disorders experienced an atmosphere of distrust in the consultation. A recently published review identified barriers to the diagnosis of MUS in primary care.^13^ The authors found that both patient communication, in which the patients’ narrative can be chaotic as GPs’ communication, in which GPs do not fully explore patients’ concerns, exhibit lack of empathy and use ineffective explanation for patients’ symptoms play a role in the physician‐patient communication which may impede the diagnosis of MUS. Further, the review describes that both GPs and patients operate within a biomedical disease model which is potentially problematic given the multi‐factorial nature of current aetiological models.^13^

This knowledge suggests a mismatch between patients’ expectations and patients’ experiences in MUS consultations. Exploring patients’ (often unmet) needs is of great importance as this contributes to a feeling of being heard as a person and probably (as a nonpecific factor) to the patient feeling better. On the other hand, not meeting patients’ expectations and a problematic communication style may reinforce catastrophizing thoughts and dysfunctional illness beliefs which may contribute to patients’ anxiety. This underlines the importance of doctor‐patient communication with attention for the patients’ expectations and is especially important in consultations with patients with MUS, as anxiety is a strong predictor for their health status and their health‐care use.^14^ Two widely used indicators to gain insight into individual patients’ health‐care needs and expectations are the significance patients adhere to specific health‐care aspects (ie, importance) and the actual experience of patients with that specific health‐care aspect (ie, performance).^15^ Expectations are defined as the extent of importance patients attach to communication aspects, and experiences are defined as the extent to which patients receive the communication aspects from their GP. As far as we know, quantitative studies focusing on the patients’ expectations and their experiences of health‐care needs have not been performed before in patients with MUS. Although there is evidence that patients with MUS differ from patients with MES regarding their expectations^5^ it is not known whether this also applies to patients’ own experiences and whether GPs communicate in a manner that meets patients’ expectations. Therefore, the first aim of this study is to explore whether patients’ expectations and experiences of the consultation differ between MUS and MES patients and to test the extent to which GPs meet patients’ expectations.

Furthermore, a review described the influence of context effects on health outcomes and found that nonspecific therapeutic elements, such as doctor‐patient communication and doctor‐patient relationship may have positive effects’ on patients’ blood pressure, symptom distress and frequency of health‐care visits.^16^ Another review described the effects of varied communication on clinical patients’ pain and found that informing patients in an empathic way with positive suggestions leads to less symptom distress.^26^ Van Dulmen et al^18^ found that the physician‐patient encounter in which GPs pay attention to empathic interaction, patient‐centredness, and diagnostic and prognostic information may have positive effects on patients’ health status, like reduction in pain and blood pressure, improvement of complaints and reduced levels of anxiety. However, it is not known whether this also applies to patients with MUS as their anxiety may be related to their potentially unmet needs and to the persistence of their symptoms. It is known that patients with MUS in general report higher rates of anxiety than patients with MES. Therefore, the GPs’ communication in the clinical encounter may have a stronger impact on reducing anxiety in MUS patients compared to MES patients. On the other hand, many GPs find MUS consultations challenging and experience communication problems during these consultations. Therefore, the GPs’ communication may have a stronger association with an increase in anxiety for MUS patients compared to MES patients. The second aim of this study is to explore the association of patients’ experiences and meeting patients’ expectations on their anxiety after the consultation. We hypothesized that patients with MUS experience GPs’ communication as less adequate than patients with MES. Moreover, we assumed that meeting patients’ expectations reduce anxiety level for both patients with MUS and MES.

## 2 METHODS

We performed a prospective cohort study based on data from questionnaires completed by patients and GPs. We studied differences between MUS and MES patients concerning what patients expected from their GP regarding communication, what they experienced during the consultation and the extent to which GPs met patients’ expectation. Next, we studied how the experienced communication and the extent to which GPs met patients’ expectations are associated with the (change in) patient's anxiety.

### 2.1 Study sample and procedure

We approached 36 GPs with different backgrounds regarding sex, age, years of work experiences and location of the practice, of whom 20 (56%) agreed to participate. Data were collected in primary care practices in the region of Nijmegen, the Netherlands, between April and September 2015. All patients who visited the GP clinic during pre‐selected study days were invited to participate, except those who did not speak Dutch well and patients under 18 years old. The consultations with participants were video‐recorded but the observation of these videos was used to examine other research questions. A researcher asked the patient before the consultation for written consent and to complete a questionnaire; after the consultation, the same questionnaire had to be filled out. The GP completed a questionnaire after each consultation, blind for the questionnaires of the patient.

### 2.2 The GP questionnaire

Immediately after each consultation, the GP answered the following question: “Do you think this patient has MUS?” on a 3‐point scale relating to the presentation of physical symptoms: (a) could not be explained by a recognizable disease (ie, MUS consultation), (b) could be explained by a recognizable disease (ie, MES consultation) or (c) could partly be explained by a recognizable disease (ie, partial MUS consultation). This latter group was excluded for all analyses, as we wanted to compare the two clearly defined groups of patients. This scale has face validity as it can easily be understood and applied by GPs during consultation hours and resembles clinical daily practice in which GPs have to interpret symptoms presented by patients as explained or unexplained by physical pathology. Previous studies in this field used the same scale.^7^, ^19^ The questionnaire included demographic information, ICPC^20^ (International Classification of Primary Care) coding of the consultation, whether the symptom was recurrent or new, the GP's management plan and the level of GP's satisfaction with the consultation on a 5‐point Likert scale.

### 2.3 The patient questionnaire

The questionnaire before the consultation included demographic data (year of birth, sex, highest level of education on a descriptive scale with 4 categories, current work status, sick leave), familiarity with the GP on a 5‐point Likert scale, whether the symptom was recurrent or new and the reason for encounter,^14^ the QUOTE‐COMM (Quality Of care Through the patients’ Eyes),^15^, ^16^ the abbreviated STAI (State‐Trait Anxiety Inventory)^17^ and the measurement of the functional health status using the COOP/WONCA^18^ (The Dartmouth Primary Care Cooperative Information/World Organization of Family Doctors). The COOP/WONCA measures a person's functional health status, that is a measurement of an individual's overall well‐being as distinct from the status of severity of their his/her problem(s). Each item of the COOP/WONCA was rated on a 5‐point ordinal scale. The reference period was 2 weeks. Seven dimensions of functional health status are assessed with the COOP/WONCA: physical fitness, being bothered by emotional problems, difficulties in performing daily activities, limitations in social activities, overall health, presence of pain and presence of fatigue. The post‐consultation questionnaire included the QUOTE‐COMM and the STAI.

### 2.4 Patients’ expectation and experiences score

Patients’ expectations and their experiences were measured using the QUOTE‐COMM.^15^, ^16^ The QUOTE‐COMM has an affect‐oriented scale of seven items in which the primary focus is on the emotional aspects of the interaction, a task‐oriented scale of 6 items in which the primary focus is on the exchange of medical content and a therapy‐oriented scale of 6 items in which the primary focus is on therapeutic aspects. Before the consultation, patients assessed how important they considered various communication aspects for the next consultation on a 4‐point Likert scale (expectations). After the consultation, patients rated the GPs’ performance of these aspects on a 4‐point Likert scale (experiences). Consequently, within communication, we distinguished task‐oriented, affect‐oriented and therapy‐oriented communication (see Appendix S1). Cronbach's α of the QUOTE‐COMM before the consultation was in our study 0.84 for the task‐oriented scale, 0.87 for the affect‐oriented scale and 0.79 for the therapy‐oriented scale meaning good internal consistency. After the consultation, these were 0.73 and 0.81 for respectively the affect‐oriented scale and therapy‐oriented scale. The Cronbach's α for the therapy‐oriented scale was after the consultation 0.56.

### 2.5 State anxiety

The state anxiety sum score was measured before and after the consultation by the abbreviated STAI (State‐Trait Anxiety Inventory) questionnaire.^17^ This questionnaire has 10 items that assess anxiety levels: the score for each item ranges from 1 to 4, with higher scores indicating a greater state of anxiety (range 1‐4). Cronbach's α in our study of the STAI questionnaire was 0.88 before the consultation and 0.91 afterwards indicating good internal inconsistency.

### 2.6 Statistical analysis

Data were analysed using IBM Statistical Package for Social Sciences (SPSS Statistics for Windows, Version 25.0. IBM Corp., Armonk, NY, USA). Due to a skewed distribution of the item GPs’ satisfaction, this item was dichotomized into somewhat/mean satisfied or (very) satisfied. Due to a skewed distribution of the item familiarity with the GP, the item familiarity with the GP was recoded into three categories: did not know the GP hardly/at all, knew the GP moderate, knew the GP (very) well. The distribution of the education level was unequal; therefore, this item was recoded into three categories: no/primary school, secondary school or high school/university. Due to a skewed distribution for all seven items of the COOP/WONCA, all of these were recoded into three new categories: (low/mean/high score for each item of the functional health status). The expectation and experience scores were calculated for the three scales task‐, affect‐ and therapy‐oriented communication. Each scale consists of 6 or 7 communication items and the expectation and experience scores were calculated as respectively the mean that patients assessed how important they considered the communication items before the consultation and the mean that patients rated the GPs’ performance of the communication items after the consultation. To calculate the extent to which GPs met patients’ expectations, we dichotomized the variable “expectation” and we combined 1 (not important) and 2 (fairly important) to one single score and combined 3 (important) and 4 (extremely important) to another single score. The variable “experience” was dichotomized as well by combining 1 (not performed) and 2 (really not performed) to one new single score and 3 (on the whole, yes) and 4 (performed) to another single score. Next, we combined the dichotomized expectations scores with the dichotomized experiences scores with four possible outcomes: not important and not performed (congruent), important and performed (congruent), not important and performed (incongruent) important and not performed (incongruent). In the congruent category, patients did experience what they expected, and in the incongruent category, patients did not experience what they had expected. The extent to which GPs met patients’ expectations was calculated as the percentage of patients with congruent experiences divided by the total number of patients. We did this for all 19 communication items separately, which was then used to generate the mean percentage for the three main scales: task‐oriented, affect‐oriented and therapy‐oriented communication. We calculated the expectation, the experience score and the extent to which GPs met patients’ expectations for task‐, affect‐ and therapy‐oriented communication. To explore differences in patients’ expectations and experiences in consultations with patients with MUS and MES and the extent to which GPs met patients’ expectations (our first aim) a *t* test, a Mann–Whitney *U* test or a chi‐squared test was used, depending on the distribution of the outcome.

For the second aim (association between patients’ experiences for task‐, affect‐ or therapy‐oriented communication of the consultation with their anxiety after the consultation), we used a linear regression model. We excluded questionnaires in which patients consecutively picked the same extreme side of the scale (ie, chose both the negatively formulated as well as the positively formulated answers, > 90%) to all question or questionnaires where <70% of the STAI questions were answered. We included anxiety before the consultation, complaint type (MUS vs MES), and experience score for task‐, affect‐ or therapy‐oriented communication as potential explanatory factors. We included the complaint type by experience score (for task‐, affect‐ or therapy‐oriented communication) interaction term as well and evaluated whether the interaction term was significant. If the coefficient on the interaction term was statistically significant, there was a difference between patients with MUS and MES in how the experience score for task‐, affect‐ or therapy‐oriented communication affected their anxiety level. As we distinguished a task‐, an affect‐ and a therapy‐oriented scale, we performed three separate regression analyses. To explore the association between the extent to which GPs met patients’ expectations for task‐, affect‐ or therapy‐oriented communication with their anxiety after the consultation, we substituted the experience score for task‐, affect‐ or therapy‐oriented communication with the extent to which GPs met patients’ expectations score for task‐, affect‐ or therapy‐oriented communication in the linear regression analysis. Again, we performed three separate regression analyses (Table 3 ).

## 3 RESULTS

**Figure 1:**
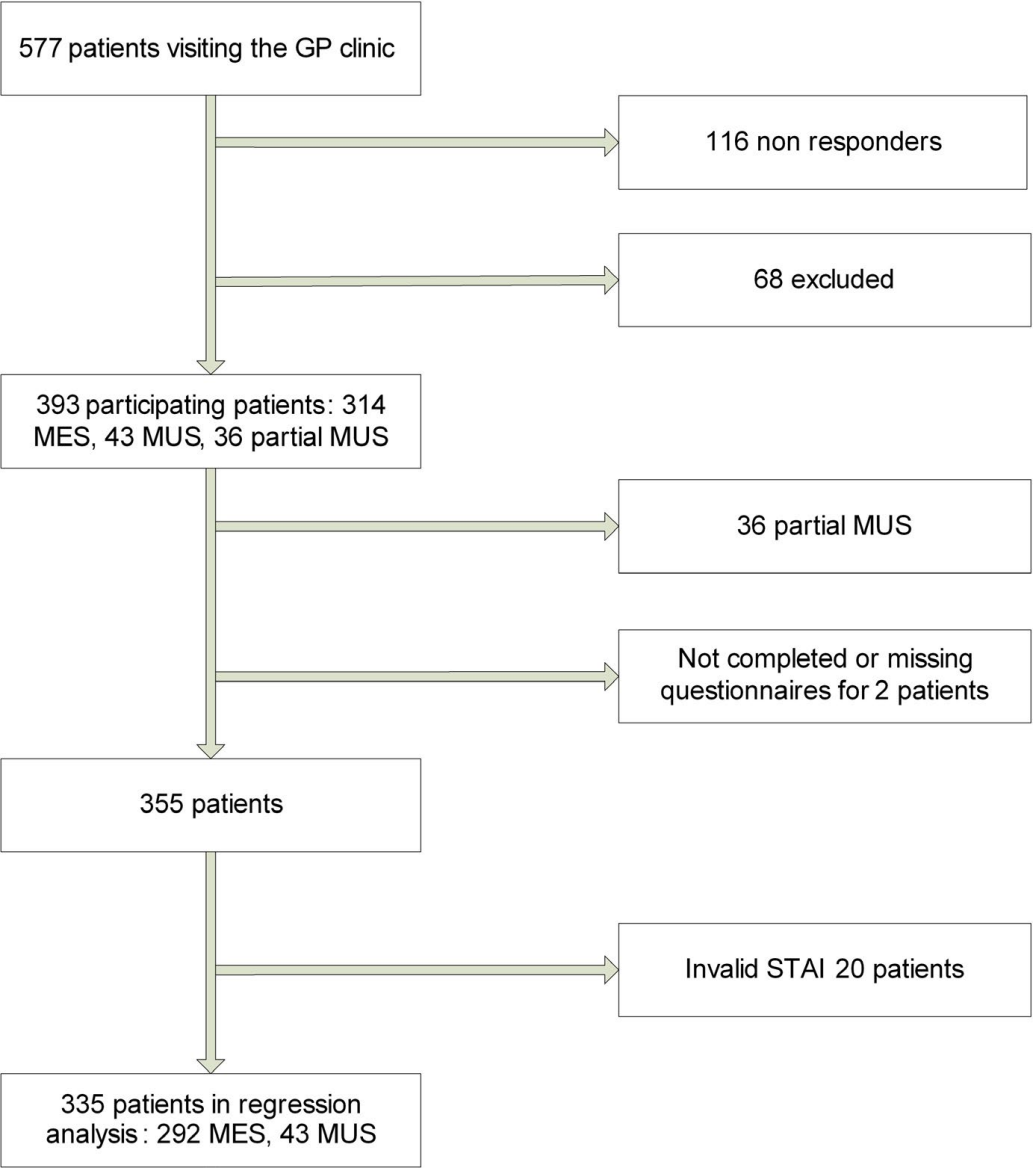
Flow chart of patients who visited their general practitioner during the study days. MES, medically explained symptoms; MUS, medically unexplained symptoms; STAI, State‐Trait Anxiety Inventory

In total, 577 patients attended their GP during the study days, of which 116 (mean age nonresponders 49.8 years, 36% men) did not want to participate and 68 were excluded, mostly because they were younger than 18 or did not speak Dutch. Of the remaining 393 patients, 43 had consultations that were labelled as MUS, 314 were labelled as MES and the other 36 had “partial MUS.” Two patients were excluded because of missing questionnaires. A total of 20, of which all were MES patients, were excluded for the regression analysis because of missing or invalid scores for anxiety level. Finally, 335 patients (292 MES and 43 MUS) were included in the regression analysis. The number of included patients per GP varied between 7 and 31; 2 GPs did not identify any MUS patient. For an overview of the patients who visited their GP during study days, see Figure 1.

Baseline characteristics of participating patients and an overview of the presented medically unexplained symptoms are shown in Table 1. Patients with MUS were younger and visited their GP more often for the same symptom. Furthermore, these patients scored significantly lower on the COOP/WONCA aspects feelings, social activities, overall health, pain and fatigue.

**Table 1:** Baseline characteristics of patients with MUS and MES

	MUS(n = 43)	MES(n = 292)	*P*‐value
Age means (SD)	50.5 (17.8)	56.7 (17.1)	**0.03** h
Familiarity with doctor (%)			
Not at all/hardly	17.5	30.5	0.17
Moderate	30.0	29.9	
(Very) well	52.5	39.5	
Highest education with diploma (%)			
No/primary school	11.6	11.4	0.72
Secondary education	58.1	52.3	
High school/University	30.2	36.4	
Repeated visit for symptoms (%)	76.7	58.9	**0.04** h
Male sex (%)	27.9	42.9	0.06
Sickness leave at present (%)	19.0	14.6	0.45
No work outside own house at present (%)	53.5	48.5	0.25
Visit at “own” GP (%)	83.7	75.5	0.24
Anxiety before consultation mean (SD, range 1‐4)	2.10 (0.57)	1.96 (0.64)	0.18
Doctors’ satisfaction with the consultation (%)			
Somewhat/mean	18.6	18.8	0.97
(Very) satisfied	81.4	81.2	
Physical fitness^a^ (%)			
(Very) heavy	44.2	47.2	0.85
Average	27.9	28.9	
(Very) light	27.9	23.9	
Being bothered by emotional problems^b^ (%)			
Not at all/little	39.5	72.9	**<0.001** h
Average	27.9	12.4	
Moderate(ly)/a lot	32.6	14.7	
Difficulties in performing daily activities^c^ (%)			
Hardly/no difficulties	44.2	60.9	0.09
Average	23.3	19.2	
Could hardly/not be done	32.6	19.9	
Limitations in social activities^d^ (%)			
Not at all/little	44.2	73.1	**<0.001** h
Average	30.2	13.0	
Moderate(ly)/a lot	25.6	14.0	
Overall health^e^ (%)			
Excellent/good	11.6	29.1	**<0.001** h
Average	20.9	33.7	
Bad/poor	67.4	37.3	
Presence of pain^f^ (%)			
No/light	19.0	48.9	**<0.001** h
Average	31.0	14.7	
Moderate/heavy	50.0	36.5	
Presence of fatigue^g^ (%)			
Not at all/little	31.0	56.8	**<0.001** h
Average	28.6	25.0	
Moderate(ly)/a lot	40.5	18.2	

MES, medically explained symptoms; MUS, medically unexplained symptoms.

The number of presented physical symptoms (n) for the MUS group was as follows: musculoskeletal pain (18), abdominal discomfort (10), tiredness (6), neurological deficit (2), shortness of breath (2), globus sensation (2) headache (1), collapse (1) and hypersensitivity syndrome (1). The number of ICPC codes divided in chapters (n) for the MES group was as follows: musculoskeletal (48), psychological (31), respiratory (31), skin (29), digestive (27), circulatory (26), ear (14), general and unspecified (13), eye (13), endocrine (13), female genital system and breast (8), urology (7), male genital system (7), social problems (7), blood, lymphatics and spleen (4), neurological (4), pregnancy and childbirth (4), unknown (6).

a Hardest physical effort during at least 2 minutes, from “very heavy” to “very light.”

b Extent of being bothered by emotional problems, from “not at all” to “a lot.”

c Extent of difficulties in doing daily activities, from “no difficulty” to “could not be done.”

d Extent to which social activity is limited by physical and emotional health, from “not at all” to “a lot.”

e Overall health, from “excellent” to “poor.”

f Presence of pain, from “no” to “heavy.”

g Presence of fatigue, from “no” to “a lot.”

h Bold values are statistically significant.

### 3.1 Patients’ expectations, patients’ experiences and meeting patients’ expectations

The expectation score, the experience score and the extent to which GPs met patients’ expectations are shown in Table 2. Patients’ expectations with regard to task‐, affect‐ and therapy‐oriented communication did not differ significantly between patients with MUS and MES. Patients presenting with either MUS or MES rated their experiences for task‐related and affect‐oriented communication of their GP higher than their expectations. However, MUS patients experienced the task‐oriented communication as slightly lower than MES patients (3.28 vs 3.47; *P* = 0.01). Furthermore, GPs met patients’ expectations on task‐oriented communication less often in patients with MUS compared to patients with MES (29.8% vs 19.1%; *P* = <0.001).

**Table 2:** Expectation score (range 1‐4), experience score (range 1‐4) and the extent to which GPs meet patients’ expectations for patient with MUS and MES

	Expectation			Experience			Meeting patients’ expectations		
	MUS mean (SD)	MES mean (SD)	*P*	MUS mean (SD)	MES mean (SD)	*P*	MUS %	MES %	*P*
Task	3.05 (0.68)	3.14 (0.58)	0.35	3.28 (0.60)	3.47 (0.62)	**0.01** a	70.2	80.9	**0.00** a *
Affect	3.25 (0.62)	3.25 (0.49)	0.46	3.88 (0.30)	3.90 (0.26)	0.56	86.2	90.8	0.13
Therapy	2.83 (0.68)	2.93 (0.59)	0.32	2.63 (0.77)	2.63 (0.72)	0.97	59.5	61.0	0.70

MES, medically explained symptoms; MUS, medically unexplained symptoms.

Within communication we distinguished task‐oriented, affect‐oriented and therapy‐oriented communication.

a Bold values are statistically significant.

* *P* = <0.01.

### 3.2 Patients’ anxiety

The mean anxiety level after the consultation was 1.84 for patients with MUS and 1.72 for patients with MES. This was for both groups significantly lower than the mean anxiety level before the consultation. There was no difference in the mean anxiety level after the consultation between patients with MUS (1.84) and MES (1.72; *P* = 0.23). There was no difference of change in anxiety between the two groups (MUS −0.26, MES −0.23, *P* = 0.62). Experiencing affect‐oriented communication had a stronger association with the post‐consultation anxiety for patients with MUS (−0.63, 95% Cl −1.07 to −0.19) than MES (Table 3). The regression coefficient of −0.63 can be interpreted as follows: for every unit increase/difference in the experience score of affect‐oriented communication, there is a −0.63 greater decrease/difference in the level of anxiety for MUS patients compared to MES patients. For the MES group, we found a regression coefficient of −0.13 (95% Cl −0.33 to 0.06).

**Table 3:** Association between post‐consultation anxiety and the explanatory factors for task‐oriented, affect‐oriented and therapy‐oriented communication

	Anxiety after the consultation					
	Task‐oriented		Affect‐oriented		Therapy‐oriented	
	β	95% CI (*P*)	β	95% CI (*P*)	β	95% CI (*P*)
Experience						
Anxiety before	0.73	0.65 to 0.82 (0.00)***	0.74	0.65 to 0.82 (0.00)***	0.75	0.66 to 0.83 (0.00)***
Group MUS	0.58	−0.14 to 1.30 (0.11)	2.35	0.64 to 4.06 (0.01)**	0.38	−0.13 to 0.88 (0.14)
MES	0		0		0	
Experience	−0.08	−0.16 to 0.00 (0.04)*	−0.13	−0.33 to 0.06 (0.17)**	−0.03	−0.10 to 0.04 (0.44)
Interaction						
MUS*Experience	−0.20	−0.41 to 0.01 (0.06)	−0.63	−1.07 to −0.19 (0.01)	−0.17	−0.35 to 0.01 (0.07)
MES*Experience	0		0		0	
Meeting patients’ expectations						
Anxiety before	0.74	0.65 to 0.83 (0.00)***	0.73	0.65 to 0.82 (0.00)***	0.74	0.65 to 0.83 (0.00)***
Group MUS	−0.01	−0.38 to 0.36 (0.97)	−0.04	−0.56 to 0.48 (0.87)	0.09	−0.25 to 0.44 (0.59)
MES	0		0		0	
Meeting patients’ expectations	−0.12	−0.34 to 0.09 (0.26)	−0.13	−0.41 to 0.16 (0.38)	−0.10	−0.30 to 0.09 (0.30)
Interaction						
MUS*meeting patients’ expectations	−0.11	−0.59 to 0.36 (0.64)	−0.05	−0.62 to 0.52 (0.86)	−0.28	−0.79 to 0.23 (0.28)
MES* meeting patients’ expectations	0		0		0	

MES, medically explained symptoms; MUS, medically unexplained symptoms.

All results are adjusted for confounders.

*P <* *0.05; ^**^ *P *<* *0.01; ^***^ *P *<* *0.001.

## 4 DISCUSSION AND CONCLUSION

### 4.1 Summary of main findings

We did not find differences between expectations of patients with MUS and MES regarding their GPs’ task‐, affect‐ and therapy‐oriented communication. Patients presenting with either MUS or MES rated their experiences for task‐related and affect‐oriented communication of their GP higher than their expectations. However, patients with MUS experienced their GPs’ task‐oriented performance significantly lower than patients with MES did. GPs met patients’ expectations on task‐oriented communication less often in patients with MUS compared to patients with MES. Furthermore, we found that experiencing affect‐oriented communication had the greatest effect in reducing anxiety for patients with MUS compared to patients with MES.

### 4.2 Comparison with existing literature

As far as we know, no quantitative studies focusing on patients’ expectations and their experiences have been performed in patients with MUS before. Furthermore, the effect of what patients experience and the extent to which GPs meet patients’ expectations during MUS and MES consultations on their anxiety levels after the consultation have not been described earlier. The finding that experiencing affect‐oriented communication had the largest effect in reducing patients’ anxiety levels with a greater effect for patients with MUS compared to patients with MES and the finding that experiencing task‐oriented communication reduced anxiety levels for both patients with MUS and MES have not been reported before.

Earlier research showed that patients with MUS sought more emotional support than patients with MES.^5^ We did not measure emotional support directly but the expectations regarding affect‐oriented communication did not significantly differ between patients with MUS and MES. Ring et al^7^ found that GPs rarely showed empathy in their verbal communication with patients. We found that patients with either MUS or MES experienced their GPs’ empathy equally high. The difference might be explained as in our study patients rated their experiences themselves, while the results in the study of Ring et al were more or less an interpretation of the researchers. The lower experience score on task‐oriented communication for patients with MUS is mainly the result of a lower score for the communicational item “GP diagnosed what was wrong,” which is understandable. Although many GPs struggle with the explanation of the origin of symptoms,^11^, ^19^, ^20^ we found that patients with either MUS or MES rated the GPs’ explanation similar. This is in line with previous research in which the authors described that patients with MUS sought no more explanation than patients with MES.^5^ Further, Salmon et al^5^ found that MUS patients did not want more somatic intervention than MES patients, and this is in accordance with our findings as we found that the expectations regarding the therapy‐oriented communication did not differ between patients with MUS and MES. In fact, we found that patients’ expectations regarding the communication element “prescribe a medicine” were significantly lower for MUS patients than for MES patients. Although previous studies reported that GPs experience difficulties in managing MUS consultations,^1^, ^7^, ^20^, ^21^, ^22^, ^23^ we found that patients with MUS rated their experiences for task‐related and affect‐oriented communication of their GP higher than their expectations. We assume that GPs’ general perception regarding managing patients with MUS concerns severe MUS patients (ie, having multiple symptoms with substantial symptom related disability and health‐care use, having a poor prognosis)^24^ while the included MUS patients in our study did probably not belong to this category of severe MUS patients as most patients with MUS and GPs did not report a high number of symptoms or the involvement of different multiple body systems or emotional comorbid disorders. As a consequence, this study possibly overestimates the patients’ experiences in MUS consultations. However, as only 2.5% belong to the patients with severe MUS,^1^ this might have a small effect on the validity of our study as we have a high sample size. Further, we found that GPs met patients’ expectations on task‐oriented communication less often for patients with MUS compared to patients with MES although the level of meeting expectations was high. Concerning the communicational items “diagnosing what is wrong” and “helping with my problem,” the expectations of MUS patients were higher than they experienced while concerning the four other communicational elements regarding task‐oriented communication their expectations were lower than they experienced.

We found no differences in change in anxiety levels between patients with MUS and MES. It is known that patients with MUS in general are associated with higher rates of anxiety than patients with diseases with comparable symptoms.^25^, ^27^ We assume that these higher rates of anxiety concerns severe MUS patients, while the included MUS patients in our study did probably not belong to these category of severe MUS patients. The benefit of affect‐oriented communication on patients’ anxiety as we found has been described previously.^16^, ^18^, ^28^, ^29^ These studies were, however, not specifically focused on patients with MUS. Van Dulmen et al^30^ found in patients with functional abdominal complaints (which can be considered MUS) that the anxiety level of significantly diminished during a series of consultations. However, Pincus et al^31^ reviewed the impact of affective and cognitive reassurance on patients’ outcomes and found that affective reassurance showed inconsistent findings. Some of the included studies showed an association between affective reassurance and higher satisfaction, while others showed that affective reassurance was associated with higher symptom burden/less improvement and lower rates of return to work. The affective reassurance included verbal and nonverbal communication like empathy, being warm and friendly. These communication items were measured in our study as well as part of the affect‐oriented communication. We found that patients with MUS benefit from affect‐oriented communication. The difference with Pincus et al may be explained from their focus mainly on reassurance, while we did not specifically focus on the reassurance but also on other communication elements.

### 4.3 Strengths and limitations

This study has a number of strengths. First, this study analysed real‐life, everyday medical visits. Second, this is the first study with patients with MUS, which examines their experiences in perspective of their expectations. Measuring patients’ experience of their consultations directly after the consultation lowers the risk of recall bias. Third, this study is unique by correlating the patients’ anxiety level to their experience, for both MUS and MES patients. However, there are also some limitations of this study, such as the missing data of the nonresponders (apart from their age and sex). Furthermore, the extent to which GPs met patients’ expectations was calculated using a dichotomized score for the expectation and experience score. Although this method is also used in another study,^16^ the cut‐off value to distinguish aspects that are (not) expected and (not) experienced to the patient is arbitrary. Furthermore, the subjective measurement of the patients’ experience could have introduced the risk of a “halo effect”: a cognitive bias in which the patient's overall impression of the GP influences the patient's feelings and thoughts about the GPs’ management. Although we measured 19 different communication aspects with the QUOTE‐COMM (see Appendix S1), we distinguished three main categories (task‐, affect‐ and therapy‐oriented communication), as previous research already revealed this distinction within the QUOTE‐COMM questionnaire.^15^, ^16^ Moreover, focussing on all 19 communication aspects individually amplifies the probability of differences just by chance. Another limitation of this study is the moderate Cronbach's α for the therapy‐oriented scale after the consultation, which means that the post‐visit therapy‐oriented communication outcomes should be interpreted with caution. We found that the communication element “referral to another specialist,” had the lowest correlation with the total score of the scale. However, removing this item would lead to a Cronbach's alpha of 0.61, a minimal improvement of the internal consistency. Therefore, we did not delete the communicational item “referral to another specialist.” We excluded the partial MUS group for all analyses, as we wanted to compare two clearly distinguished groups. In case, we should merge patients with partial MUS and patients with MUS together as one common MUS group, this would perhaps have resulted into less clear outcomes. Another limitation of this study might be the selection of the participating GPs. Although we tried to reduce this bias by selecting GPs with different (clinical) backgrounds we assume that the not participating GPs are less interested in MUS or have more negative attitudes and thus experience more and other problems during the consultations. As a consequence, this study possibly overestimates the patients’ experiences in MUS consultations. Furthermore, we could also use a multilevel model instead of a linear regression model. However, we found low ICC of the random factor GP for all outcomes, which suggest a minimal clustering of the data. Therefore, a multilevel model would not show other results as we found by using a linear regression model. Finally, in contrast to many other studies,^2^, ^3^ we identified patients as MUS who had in the doctor's opinion unexplained symptoms and not for example based on duration of symptoms or other criteria. This might have induced a large inter‐doctor variation of labelling patients with MUS.

### 4.4 Practical implications

Increasing insight and paying attention to individual patients’ expectations may enhance GPs’ communication. This is even more important for patients with MUS, as meeting their expectations may contribute to lower anxiety levels. GPs’ communication in MUS consultations should focus on task‐ and in particular on affect‐oriented communication, as this is associated with lower anxiety levels of these specific patients. Further research should focus on developing and implementing a communication intervention that optimizes communication with patients with MUS. This communication intervention should focus on the consultation itself, without disturbing the normal flow of the consultation. A clinical assessment (history taking, physical examination, request of additional testing, explanation of what is wrong, and advice) of symptoms and nonspecific therapeutic elements (such as expectations, positive communication, empathy and support) during the consultation process may guide towards an effective, acceptable and feasible treatment strategy for patients with MUS in primary care.

## 5 CONCLUSION

Many GPs think that patients with MUS differ from other patients because they want more explanation and somatic interventions. However, as we found that patients’ expectations do not differ between patients with MUS and MES, GPs should reflect on these assumptions. GPs’ communication training should focus on a thorough self‐reflection and should pay attention to task‐ and especially affect‐oriented communication as these are associated with reduced levels of anxiety.

## CONFLICT OF INTEREST

The authors declare that they have no conflict of interest.

## ETHICS COMMITTEE AND INFORMED CONSENT

The study was carried out according to Dutch privacy legislation. The privacy regulations were approved by the Dutch Data Protection Authority. The research ethics committee of the Radboud University Nijmegen Medical Center concluded that the study could be carried out in accordance with the applicable rules in the Netherlands (file number 2015‐1566). Written informed consent was obtained from all participating patients; patients were able to withdraw their consent at any time.
