# Supplementary material for: The association between patients’ expectations and experiences of task‐, affect‐ and therapy‐oriented communication and their anxiety in medically unexplained symptoms consultations
Source: Health Expect. 2018 Dec 30;22(3):338–47. doi: 10.1111/hex.12854 (PMC6543164; doi:10.1111/hex.12854)
Supplement: Supplementary file 1 [file HEX-22-338-s001.doc]

**Appendices**

*Appendix S1.*

*QUOTE-COMM (Quality Of communication Through the patients’ Eyes) before the consultation.*

**I think it is important that the doctor:**

**1=not important 2=fairly important 3= important 4=extremely important**

| **Task oriented aspects**  Examines me  Diagnoses what’s wrong  Explains well what’s wrong  Gives advice on what to do  Helps me with my problem  Informs me well about the treatment  **Affect oriented aspects**  Is friendly  Takes my problem seriously  Listens to me well  Is open to me  Takes enough time for me  Is empathic to me  Gives me enough attention  **Therapy oriented aspects**  Takes the final decision about the treatment  Prescribes medication  Discusses different treatment options  Referrers me to another specialist  Involves me in taking a decision  Informs me about side effects | 1 2 3 4  1 2 3 4  1 2 3 4  1 2 3 4  1 2 3 4  1 2 3 4  1 2 3 4  1 2 3 4  1 2 3 4  1 2 3 4  1 2 3 4  1 2 3 4  1 2 3 4  1 2 3 4  1 2 3 4  1 2 3 4  1 2 3 4  1 2 3 4  1 2 3 4 |
| --- | --- |

*QUOTE-COMM (Quality Of communication Through the patients’ Eyes) after the consultation.*

**1=not 2=really not 3=on the whole, yes** 4= yes

| **Task oriented aspects**  The doctor examined me  The doctor diagnosed what was wrong  The doctor explained well what was wrong  The doctor gave advice on what to do  The doctor helped me with my problem  The doctor informed me well about the treatment  **Affect oriented aspects**  The doctor was friendly  The doctor took my problem seriously  The doctor listened well to me  The doctor was open to me  The doctor took enough time for me  The doctor was empathic to me  The doctor gave me enough attention  **Therapy oriented aspects**  The doctor took the final decision about the treatment  The doctor prescribed medication  The doctor discusses different treatment options  The doctor referred me to a specialist  The doctor involved me in taking a decision  The doctor informed me about side effects | 1 2 3 4  1 2 3 4  1 2 3 4  1 2 3 4  1 2 3 4  1 2 3 4  1 2 3 4  1 2 3 4  1 2 3 4  1 2 3 4  1 2 3 4  1 2 3 4  1 2 3 4  1 2 3 4  1 2 3 4  1 2 3 4  1 2 3 4  1 2 3 4  1 2 3 4 |
| --- | --- |
